# Intravitreal dexamethasone implants versus intravitreal anti-VEGF treatment in treating patients with retinal vein occlusion: a meta-analysis

**DOI:** 10.1186/s12886-018-1016-7

**Published:** 2019-01-08

**Authors:** Lixiong Gao, Lijun Zhou, Chunyu Tian, Na Li, Weiyang Shao, Xiujun Peng, Qian Shi

**Affiliations:** 1 Ophthalmology Department, General Navy Hospital of Chinese People’s Liberation Army, Beijing, 100048 China; 2 Central Laboratory, General Navy Hospital of Chinese People’s Liberation Army, Beijing, 100048 China

**Keywords:** Dexamethasone intravitreal implant, Anti-VEGF treatment, Retinal vein occlusion, Meta-analysis

## Abstract

**Background:**

Retinal vein occlusion (RVO) is a common retinal venous disorder that causes vision loss. No specific therapy has been developed. Controversy exists regarding two treatments: intravitreal dexamethasone implants and anti-vascular endothelial growth factor (VEGF). The goal of this study is to compare the effectiveness and safety of dexamethasone implants and anti-VEGF treatment for RVO.

**Methods:**

The PubMed, Embase, and Cochrane Library databases were searched for studies comparing dexamethasone implants with anti-VEGF in patients with RVO. Best-corrected visual acuity (BCVA), central subfield thickness (CST), intraocular pressure changes, conjunctival haemorrhage, reduced VA, and macular oedema were extracted from the final included studies. RevMan 5.3 was used to conduct the quantitative analysis and bias assessment.

**Results:**

Four randomised controlled trials assessing 969 eyes were included. The anti-VEGF treatment showed better BCVA improvement (mean difference [MD] = − 10.59, *P* < 0.00001) and more CST decrease (MD = − 86.71 μm, *P* = 0.02) than the dexamethasone implants. However, the dexamethasone implants required fewer injections. As for adverse effects, the dexamethasone implants showed significantly higher intraocular pressure (IOP) and more cataracts than the anti-VEGF treatment. No significant differences were found in conjunctival haemorrhage, reduced VA, and macular oedema.

**Conclusions:**

Anti-VEGF treatment showed better functional and anatomical improvement with less risk of IOP elevation and cataract formation compared to dexamethasone implants. Thus, anti-VEGF treatment is the first choice for treating RVO patients.

## Background

Retinal vein occlusion (RVO), including branch retinal vein occlusion (BRVO) and central retinal vein occlusion (CRVO), is a common retinal venous disorder that can lead to variable visual impairment [1]. BRVO is defined as the occlusion of any retinal vein branch draining a portion of the retina. CRVO is an occlusion of the central retinal vein that results in four-quadrant retinal involvement. Currently, hypertension, hyperlipidaemia, arteriosclerosis, and diabetes are known common risk factors for RVO [2]. As systemic vascular disorders are on the rise all over the world, more patients will be diagnosed with RVO in the future.

The pathogenesis of BRVO is more likely to involve retinal vein compression [3], where the artery crosses over a vein [4]. The pathogenesis of CRVO is not fully understood. The central retinal artery, which shares the same adventitial sheath with the adjacent central retinal vein, may compress the vein and lead to occlusion, especially in eyes with increased arterial rigidity from hypertension and arteriosclerosis [5]. Nevertheless, both BRVO and CRVO share an identical pathology, an increase in the intravascular pressure in the obstructed vein and damage to the vessel wall, which result in fluid leakage and the release of inflammatory cytokines such as vascular endothelial growth factor (VEGF), respectively [6]. As these pathological characteristics always lead to macular oedema and vitreous haemorrhage secondary to neovascularization of the retina, vision loss in RVO patients is mainly caused by these two conditions [7].

As inflammation after the obstruction of the retinal vein is common in RVO, intravitreal corticosteroid injection has been shown to improve visual acuity (VA) and foveal thickness [1]. However, the improvements in VA were limited to the first and second months [8]. Decreasing corticosteroid concentrations are believed to be the causes. A sustained-delivery biodegradable dexamethasone (DEX) intravitreal implant (Ozurdex; Allergan Plc, Dublin, Ireland) that releases corticosteroid for up to 6 months after intravitreal injection has been developed and has proven to be safe and effective in both BRVO and CRVO patients [9–11]. However, fluctuations in VA and steroid-related complications such as elevated intraocular pressure (IOP) and cataracts were observed [11].

A marked rise in intravitreal levels of VEGF was observed in eyes with RVO, which has been shown to correlate with the severity of clinical findings [6, 12]. As VEGF may cause capillary endothelial cell proliferation, anti-VEGF treatment can enhance blood flow, lower intravenous pressure, and normalise venous diameter and tortuosity, resulting in both the anatomical and functional recovery of RVO patients [13]. In June 2010, 0.5-mg ranibizumab was approved to treat RVO patients based on the results of the CRUISE study [14]. In addition, no significant difference was found in the VA changes of RVO patients between the two different anti-VEGF treatments (bevacizumab and ranibizumab) [15, 16], suggesting that the current types of anti-VEGF treatment do not affect the final results.

Many separate clinical trials have shown the effectiveness of both DEX implants and anti-VEGF treatment. Previous meta-analyses either concluded the effectiveness of monotherapy or conducted indirect comparisons [17–19]. To directly compare the final outcomes of these two treatments, head-to-head randomised controlled trials (RCTs) were recently conducted [20–23]. To date, there have been no systematic reviews involving a direct comparison of intravitreal DEX implants and anti-VEGF injection in RVO. Therefore, this meta-analysis was conducted to quantify the main outcomes and adverse effects of RVO treatment.

## Methods

### Search strategy

**Fig. 1:**
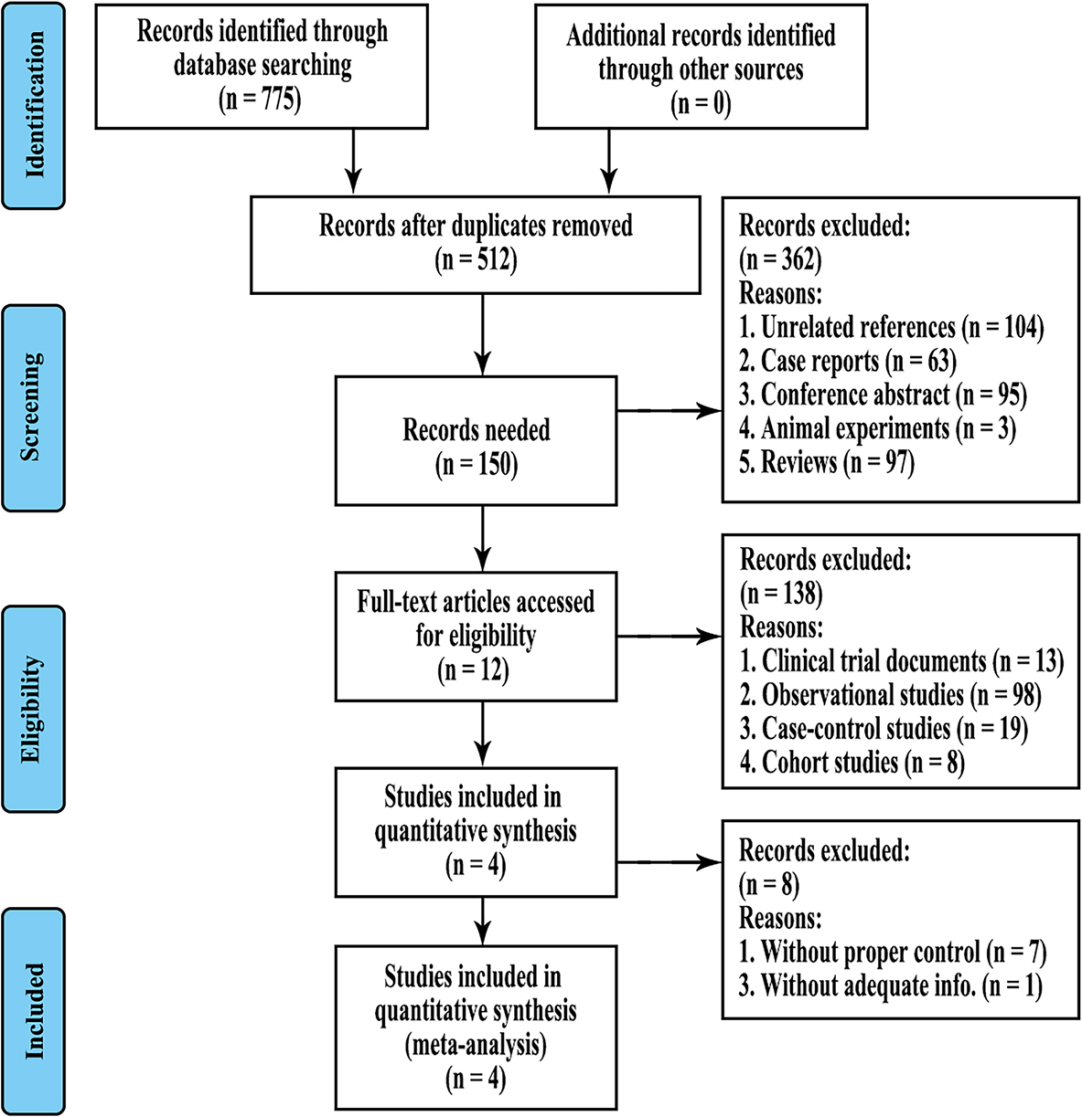
Flow chart of the literature search

This study was conducted according to the Cochrane Handbook for Systematic Reviews and Meta-Analysis (PRISMA) guidelines [24]. The PubMed, Embase, and Cochrane Library databases were screened up to August 2018 to complete the study. Keywords for DEX intravitreal implants included dexamethasone intravitreal implant and dexamethasone implant and Ozurdex. Keywords for RVO included retinal vein occlusion; retinal vein occlusions; retinal vein thrombosis; retinal vein thromboses; retinal vein obstruction and RVO. To maximise the search accuracy and avoid missing articles, terms for anti-VEGF treatment were not particularly confined since there were different types related to this method. Suitable articles were filtered and included. The literature selections are shown in the PRISMA flow diagram in Fig. 1.

### Inclusion and exclusion criteria

Studies were considered eligible in accordance with the following criteria: (1) the study design was RCT, (2) the study population included patients with RVO, (3) DEX implants and anti-VEGF treatment were both included as interventions, (4) there was a comparison between the DEX implants and the anti-VEGF groups, and (5) the observation duration lasted at least 6 months. This study excluded (1) case reports, observational studies, case control studies, cohort studies, reviews, comments, and conference abstracts and (2) studies with insufficient information.

The following primary outcomes were taken into consideration: (1) the mean BCVA change from baseline was obtained using the Early Treatment Diabetic Retinopathy Study (ETDRS); (2) the mean central subfield thickness (CST) was measured using optical coherence tomography; and (3) the number of patients with improvement in reading letters over 15. Additional adverse effects included the following: mean IOP, cataracts, conjunctival haemorrhage, macular oedema, and reduced VA. This study was designed to have no preference for the type of anti-VEGF treatment.

### Data extraction and bias assessment

Using a standard data extraction form, the relevant data were independently extracted by two reviewers (Tian and Shao). Apart from the aforementioned primary outcomes, the following factors were extracted: first author(s), publishing date, locations of study, study design, type of disease, sample size, intervention details, number of treatments, age, sex, and follow-up periods. Continuous data are formatted as mean ± standard deviation (SD). The number of related events was recorded in the discontinuous data. The formula SD = SE*√N was used to calculate the SD if only the standard error (SE) was reported. The GRADE profiler was used to assess the quality of outcomes. The Cochrane Collaboration’s tool was used to assess the risk of bias in each study based on the Cochrane Handbook.

### Statistical analysis

RevMan 5.3 was used to conduct the data statistics and meta-analysis. The different types of RVO were not distinguished during the main quantitative synthesis but were compared in the subgroup analysis. The odds ratios (OR) and mean differences (MD) were used to calculate the dichotomous data and continuous variable data with 95% confidence intervals [CI], respectively. The heterogeneity of the studies was accessed using the chi-squared test based on the values of P and I^2^. All of the meta-analyses were carried out under the random effects model. I^2^ results between 50 and 100% were considered to present heterogeneity. *P* values < 0.05 were considered statistically significant. The BCVA in the two subgroups (BRVO and CRVO) were analysed in the subgroup analysis.

## Results

### Study characteristics

Overall, 775 studies were identified (PubMed = 260, Embase = 436, and Cochrane Library = 79) up to August 2018. After removing 263 duplications, 104 unrelated articles, 3 animal studies, 95 conference abstracts, 63 case reports, and 97 reviews, 150 studies then proceeded to the screening procedure. Upon reading the titles and abstracts, we further eliminated 13 clinical trial documents, 98 observational studies, 19 case-control studies, and 8 cohort studies. After reading the full text of the remaining 12 articles, 8 records were excluded due to lack of proper controls and adequate information (Fig. 1). Four studies were further included in the quantitative synthesis.

**Fig. 2:**
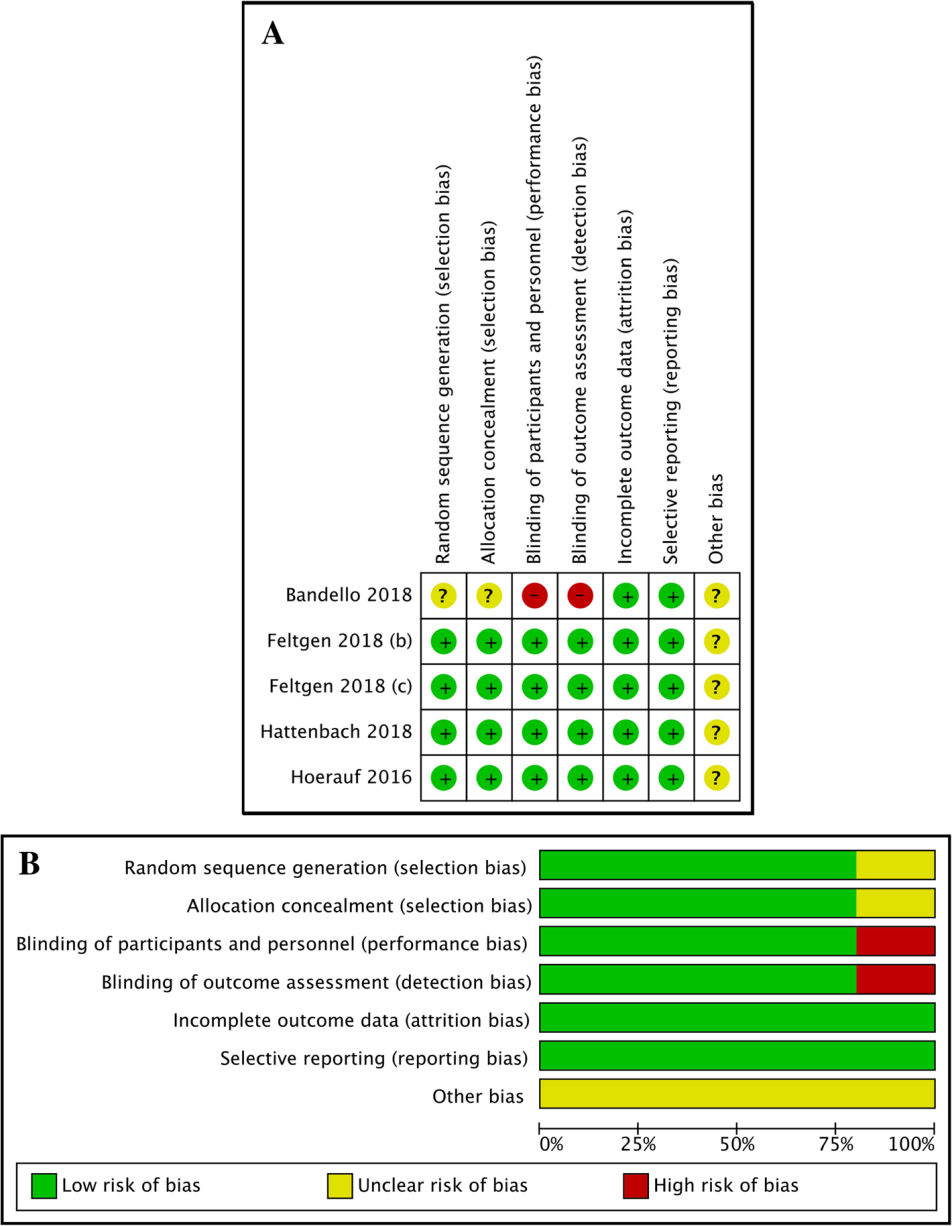
Assessment of the risk of bias in the included studies. **a**: Risk of bias summary: the authors’ judgements on each risk of bias item for the included studies. +: low risk of bias; −: high risk of bias; and?: unclear risk of bias. **b**: Risk of bias graph: authors’ judgements regarding each risk of bias item displayed as percentages across the included studies

All four included studies were RCTs that contained 969 eyes in total. The characteristics of the included studies are presented in Table 1. Two studies focused on BRVO. One study focused on CRVO. One study contained both BRVO and CRVO results. As the data were provided separately in this study, we split it into two independent studies by Feltgen (b) and Feltgen (c) (b for BRVO and c for CRVO). All four studies were published after 2016 (2016: one study; 2017: two studies; and 2018: one study). Coincidently, the treatments in all four studies were the same: 0.5 mg ranibizumab and 0.7 mg Ozurdex for the anti-VEGF drugs and DEX implants, respectively. For the anti-VEGF treatment, Hoerauf et al. and Hattenbach et al. both administered ranibizumab monthly for 3 consecutive months followed by pro re nata (PRN) treatment while Feltgen et al. directly administered PRN ranibizumab treatment. Bandello et al. administered ranibizumab monthly for 5 consecutive months followed by PRN treatment (Table 1). For the DEX implant treatment, Hoerauf et al. and Hattenbach et al. both administered Ozurdex as the initial treatment followed by monthly PRN injection. Feltgen et al. directly administered one-dose Ozurdex as the initial or later treatment, followed by PRN treatment at a time interval of no less than 5 months. Bandello et al. administered Ozurdex injection at both the first and fifth months followed by PRN injection at the tenth and eleventh months (Table 1). The risk of bias assessment is presented in Fig. 2.

**Table 1:** Characteristics of the included studies

Study	Year	Country	Type	Disease	Participant number	Intervention details	Number of treatment	Age	Sex number	BCVA at baseline	CST at baseline	Follow up duration
Bandello	2018	France, Germany, Israel, Italy, Spain, and the United Kingdom	RCT	BRVO	Total = 307 n(IVR) = 153 n(DEX) = 154	IVR: 0.5 mg monthly given for 5 consecutive months than PRN DEX: 0.7 mg Ozurdox injection at month 1 and 5; PRN on month 10 or 11	IVR: 8.0 DEX: 2.5	IVR: 65.5 ± 12.0 DEX: 68.4 ± 10.6	IVR: M: 87; F: 67 DEX: M: 92; F: 61	IVR: 59.2 ± 10.9 DEX: 56.6 ± 10.9	IVR: 544 ± 168 DEX: 547 ± 163	12
Feltgen	2018	German	RCT	BRVO	Total = 92 n(IVR) = 52 n(DEX) = 40	IVR: 0.5 mg PRN injection DEX: 0.7 mg Ozurdox 1 dose given at the initial treatment or later; time interval of PRN is at least 5 months	IVR: 4.46 ± 2.09 DEX: 0.40 ± 0.50	IVR: 64.5 ± 9.7 DEX: 64.6 ± 9.9	IVR: M: 18; F: 34 DEX: M: 22; F: 18	IVR: 56.8 ± 10.0 DEX: 58.3 ± 10.8	IVR: 563.3 ± 170.0 DEX: 547.3 ± 178.9	6
Feltgen	2018	German	RCT	CRVO	Total = 83 n(IVR) = 61 n(DEX) = 22	IVR: 0.5 mg PRN injection DEX: 0.7 mg Ozurdox 1 dose given at the initial treatment or later; time interval of PRN is at least 5 months	IVR: 3.92 ± 2.64 DEX: 0.45 ± 0.51	IVR: 66.6 ± 10.1 DEX: 61.1 ± 11.0	IVR: M: 31; F: 30 DEX: M: 30; F: 8	IVR: 54.1 ± 15.8 DEX: 53.2 ± 16.1	IVR: 698.8 ± 228.6 DEX: 721.2 ± 231.1	6
Hattenbach	2018	Germany, Great Britain, the Czech Republic, Poland and Hungary	RCT	BRVO	Total = 244 n(IVR) = 126 n(DEX) = 118	IVR: 0.5 mg monthly given for 3 consecutive months than PRN DEX: 0.7 mg Ozurdox given at the initial treatment and monthly shame PRN injection	IVR: 4.71 DEX: 1	N/A	N/A	N/A	N/A	6
Hoerauf	2016	Germany, Great Britain, Poland, and Hungary	RCT	CRVO	Total = 243 n(IVR) = 124 n(DEX) = 119	IVR: 0.5 mg monthly given for 3 consecutive months than PRN DEX: 0.7 mg Ozurdox given at the initial treatment and monthly shame PRN injection	IVR: 4.52 DEX: 1	IVR: 65.3 ± 11.4 DEX: 66.9 ± 12.4	IVR: M: 72; F: 52 DEX: M: 73; F: 46	IVR: 51.7 ± 16.5 DEX: 51.5 ± 15.6	IVR: 723.8 ± 245.9 DEX: 705.2 ± 231.1	6

### Meta-analysis

#### Mean BCVA changes

**Fig. 3:**
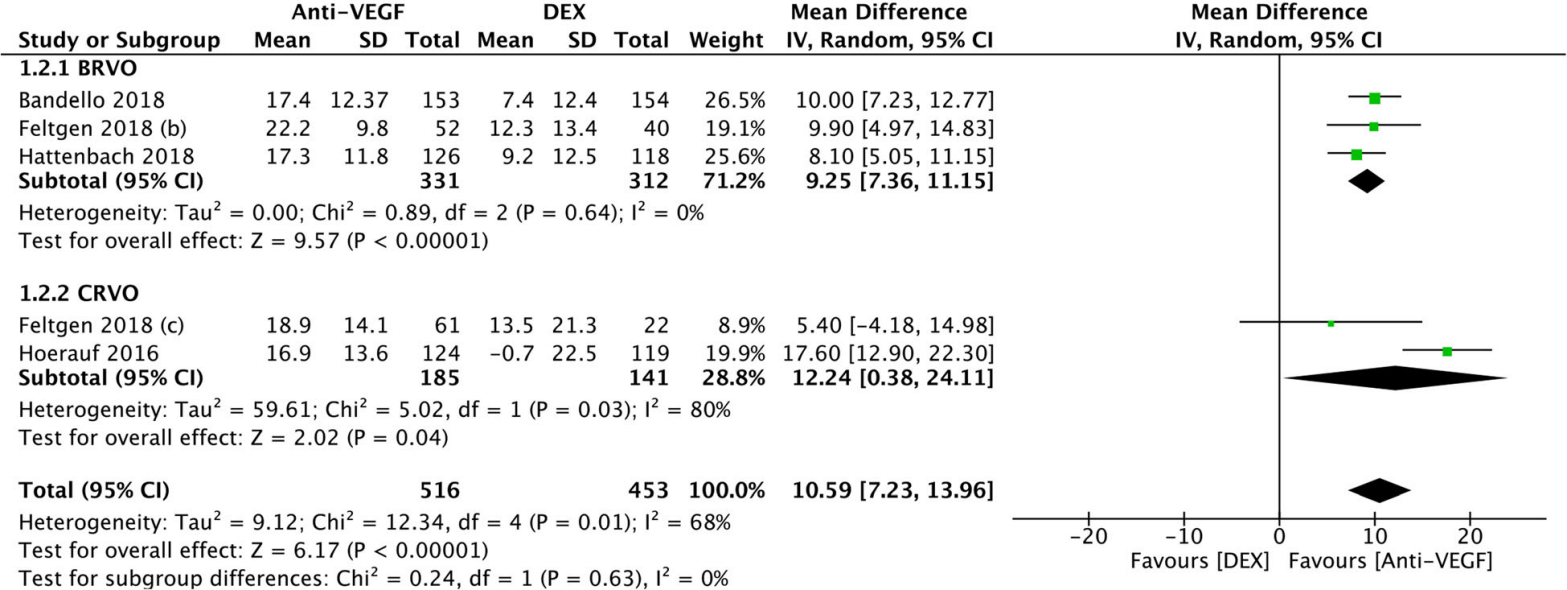
A forest plot diagram comparing the main outcome of the DEX implant with the anti-VEGF treatment at the endpoint. The results present the mean BCVA changes in RVO and the subgroup analysis of BRVO and CRVO

Hoerauf et al. and Hattenbach et al. reported the BCVA changes over 6 months. Bandello et al. reported the BCVA changes over 12 months. Feltgen (b) et al. and Feltgen (c) et al. provided extensions of both Hattenbach et al. and Hoerauf et al. with 6-month time intervals. Since the observation points and study designs were different, we synthesised all five parts of the research outcomes at their endpoints. There was a significant difference in the treatment effect on the BCVA changes. The MD of VA of the five trials was 10.59 (95% CI: 7.23 to 13.96, *P* < 0.00001, Fig. 3). Moderate heterogeneity was found (chi^2^ = 12.34, *P* = 0.01, I^2^ = 68%). To further analyse the two different types of RVO, we performed a subgroup analysis of BRVO and CRVO. The MD in the BRVO subgroup was 9.25 (95% CI: 7.36 to 11.15, *P* < 0.00001, Fig. 3). No heterogeneity was found (chi^2^ = 0.89, *P* = 0.64, I^2^ = 0%). The MD in the CRVO subgroup was 12.24 (95% CI: 0.38 to 24.11, *P* = 0.04, Fig. 3). High significant heterogeneity was found (chi^2^ = 5.02, *P* = 0.03, I^2^ = 80%). Compared to the DEX group, both the total and subgroup results showed significantly better BCVA outcomes in the anti-VEGF treatment group.

#### Significant improvement in BCVA

**Fig. 4:**
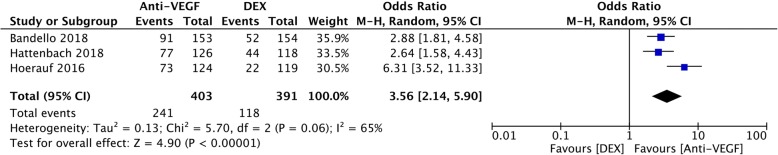
A forest plot diagram comparing the significant improvement in BCVA after the DEX implants and the anti-VEGF treatment at the endpoint

The proportion of patients who achieved significant improvement in BCVA, defined as ≥15 letters gained, was further compared. Three studies referring to this outcome were included in the quantitative synthesis. There was a significant difference in the number of patients who gained VA ≥ 15 letters. The OR of the three compared trials was 3.56 (95% CI: 2.14 to 5.90, *P* < 0.00001, Fig. 4). Moderate heterogeneity was found (chi^2^ = 5.70, *P* = 0.06, I^2^ = 65%). Anti-VEGF treatment provided more patients with significant improvements in BCVA.

#### Mean changes in central subfield thickness (CST)

**Fig. 5:**
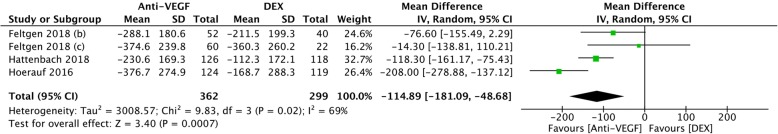
A forest plot diagram comparing the central subfield thickness after the DEX implants and the anti-VEGF treatment at the endpoint

Apart from functional improvement, anatomical relief should also be considered. Data from three studies (four researchers) assessing 661 eyes reported the mean change in CST from the baseline. The results of the quantitative synthesis showed a significantly greater reduction in the anti-VEGF groups. The MD in the CST changes of the four trials was − 114.89 (95% CI: -181.09 to − 48.68, *P* = 0.0007, Fig. 5), which means the anti-VEGF treatment better reduced the CST than the DEX implants. Moderate heterogeneity was found (chi^2^ = 9.83, *P* = 0.02, I^2^ = 69%).

#### Mean changes in IOP

**Fig. 6:**
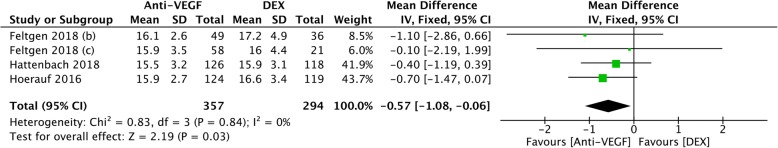
A forest plot diagram comparing the intraocular pressure changes after the DEX implants and the anti-VEGF treatment at the endpoint

As DEX is a type of glucocorticoid that causes IOP elevation in patients under long-term treatment, three articles (four studies) demonstrated changes in the IOP measurements at the endpoints. There was a significant difference between the DEX implants and the anti-VEGF treatment group, although the difference is small. The MD in the IOP change in the four trials was − 0.57 (95% CI: -1.08 to − 0.06, *P* = 0.03, Fig. 6). No heterogeneity was found in the meta-analysis (chi^2^ = 0.83, *P* = 0.84, I^2^ = 0%).

#### Adverse events

**Fig. 7:**
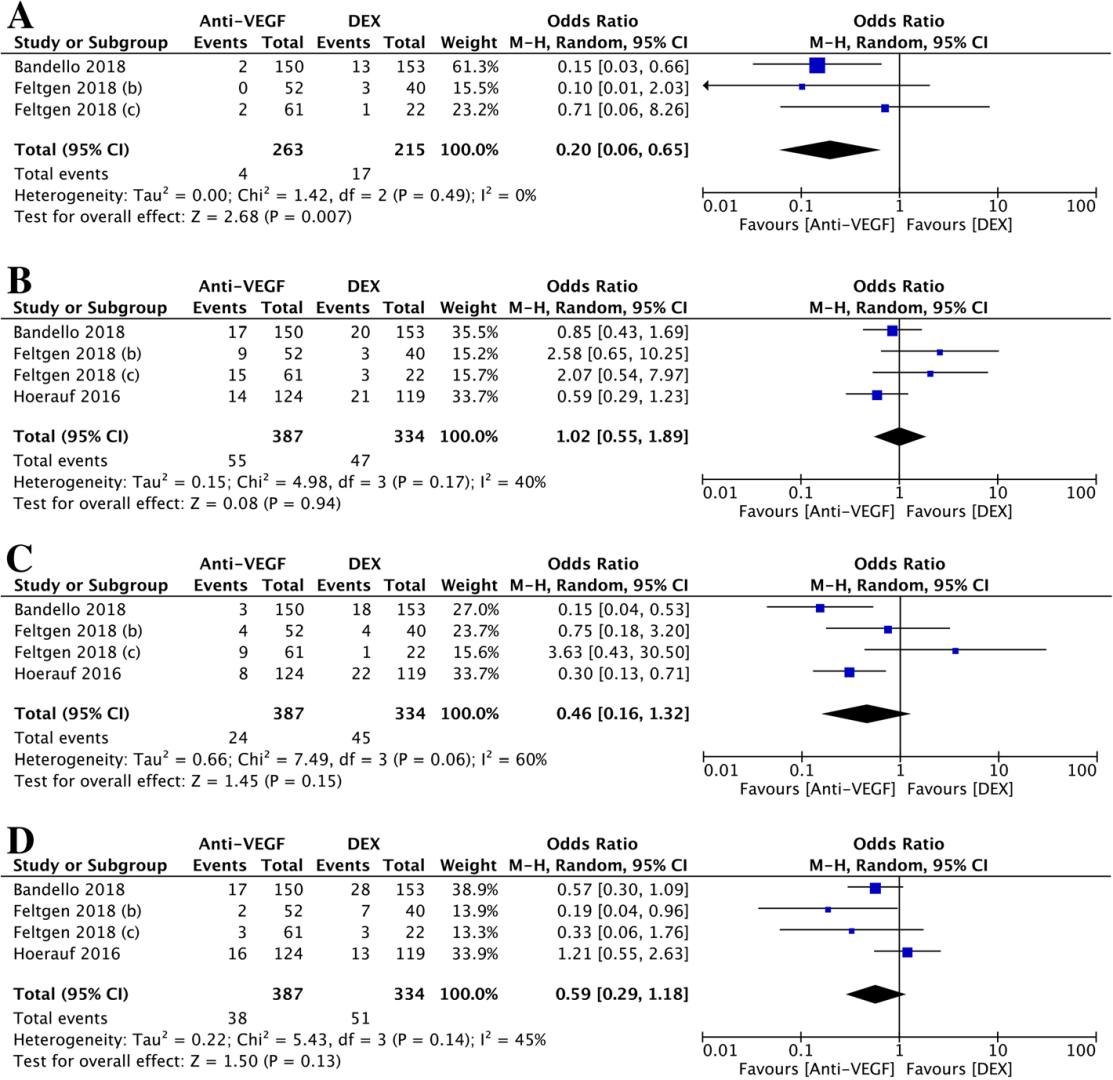
A forest plot diagram comparing the 4 main adverse effects after the DEX implant and the anti-VEGF treatment at the endpoint. **a**: Cataracts. **b**: Macular oedema. **c**: Reduced VA. **d**: Conjunctival haemorrhage

With the exception of Hattenbach et al., three articles (four studies) provided tables of the number of patients who had adverse events. Cataracts, macular oedema, reduced VA, and conjunctival haemorrhage were included in the meta-analysis. For cataracts, there was a significant difference in the number of cataract patients between the DEX implant and anti-VEGF treatment groups. The OR in the three trials was 0.20 (95% CI: 0.06 to 0.65, *P* = 0.007, Fig. 7 a). No heterogeneity was found in the meta-analysis (chi^2^ = 1.42, *P* = 0.49, I^2^ = 0%). For macular oedema, there was no significant difference between the DEX implants and the anti-VEGF treatment group. The OR in the four trials was 1.02 (95% CI: 0.55 to 1.89, *P* = 0.94, Fig. 7 b). Low heterogeneity was found in the meta-analysis (chi^2^ = 4.98, *P* = 0.17, I^2^ = 40%). For the reduced VA, there was no significant difference between the DEX implants and the anti-VEGF treatment group. The OR in four trials was 0.46 (95% CI: 0.16 to 1.32, *P* = 0.15, Fig. 7 c). Moderate heterogeneity was found in the meta-analysis (chi^2^ = 7.49, *P* = 0.06, I^2^ = 60%). For conjunctival haemorrhage, there was no significant difference between the DEX implants and the anti-VEGF treatment group. The OR in the four trials was 0.59 (95% CI: 0.29 to 1.18, *P* = 0.13, Fig. 7 d). Low heterogeneity was found in the meta-analysis (chi^2^ = 5.43, *P* = 0.14, I^2^ = 45%).

## Discussion

This study evaluated four RCTs to compare the efficacies of DEX implants and anti-VEGF treatment of RVO. Significant functional and anatomical improvement was found in both DEX implants and anti-VEGF treatment. However, anti-VEGF treatment showed significantly better BCVA improvement than DEX implants as reflected by both the better mean change of BCVA and more patients with ≥15 letters gained. There was also significantly better CST relief after anti-VEGF treatment compared to DEX implants, which coincided with the functional improvements. As for the adverse effects, there was no significant difference in conjunctival haemorrhage, macular oedema, and reduced VA between the two groups. Nevertheless, DEX implants presented significantly higher risk of IOP elevation and cataract development compared to anti-VEGF treatment. As for the RVO subtypes, BRVO and CRVO both presented similar effects, illustrating the identical pathological processes of these two RVO subtypes and the identical effects of these treatments.

A network meta-analysis comparing the different interventions related to BRVO showed better results for the anti-VEGF treatments than the DEX implants [25]. Another meta-analysis comparing intravitreal bevacizumab with triamcinolone acetonide in RVO also confirmed the better therapeutic effects of the anti-VEGF treatment [26]. A more recent meta-analysis compared the anti-VEGF treatments with corticosteroids or laser therapy for RVO. Although an indirect comparison was performed, the results indicated that the anti-VEGF treatment showed better and longer efficacy than the other remedies [18].

The current results may seem overwhelming. The anti-VEGF treatment showed better effects in almost every aspect than the DEX implants. Based on the sustained release of DEX, the effective time of the DEX implants could last for 6 months. The studies treated patients with only one dose initially or with a second injection after at least 5 months. However, after the DEX implant injection, all of the studies showed improvement in BCVA in the first 2 months [20, 22, 23], while BCVA later gradually decreased. However, the anti-VEGF treatment was carried out in a 3 + PRN model, which could create relatively more sustained VA improvements. The origin of the differences should refer to the pathology of RVO.

Assessment of the vitreous fluid in patients with RVO has confirmed the elevation of proinflammatory mediators and the decrease in anti-inflammatory cytokines [27, 28]. Macular oedema was the consequence of prolonged inflammatory states [29]. As corticosteroids can inhibit numerous local inflammatory modulators, including VEGF, and may decrease oedema through the stabilisation of vascular permeability [30], dexamethasone theoretically becomes the candidate for RVO treatment. DEX implants were evaluated in the GENEVA trial for safety and efficacy in both BRVO and CRVO [31]. The results showed identical patterns with the current included studies, an improvement in BCVA within the first 2 months and a gradual decrease over the subsequent 4 months. Nevertheless, the pharmacokinetics of DEX implants presented a trend that the concentration of DEX in the vitreous humour rose within the first 2 months and started to decrease from the third month [9], corresponding to the change in BCVA. While VEGF is widely known for its main characteristics in retinal angiogenesis, studies have found that it permits leukocyte infiltration into the retina, which is considered the key initial step in the inflammatory response [32, 33]. After treatment with corticosteroids and anti-VEGF, the VEGF concentrations in the vitreous humour decreased [30, 34]. However, the anti-VEGF treatment showed strikingly different pharmacokinetics than the DEX implants. One study found that the intravitreal half-life of bevacizumab, ranibizumab, and aflibercept in rabbits is 4.32, 2.6, and 4.7 days, respectively [34]. The data in humans have not been obtained. But the estimated intravitreal half-life of bevacizumab, ranibizumab, and aflibercept in humans is 3, 6, and 9 days, respectively [34], which highlights the foundation of continuous therapy for anti-VEGF treatment. Taken together, the decrease of the effect in the DEX implants is mainly due to the decrease in its concentration, while the anti-VEGF treatment maintains its effect due to continuous injection.

Of note, as both DEX implants and ranibizumab are expensive, the benefits and costs should be considered. According to a study focusing on the economics of CRVO, treatment via a DEX implant consumes 1 injection and saves 1.2 lines of VA, while ranibizumab consumes 8.7 injections on average and saves 2.82 lines of VA. Taking the price into consideration, the “dollars per line saved” of DEX and ranibizumab are $1961 and $7611, respectively [35]. Thus, ophthalmologists should consider various strategies when facing patients with uneven economic conditions.

Higher chances of IOP elevation was found after DEX implants [31]. The current meta-analysis also showed a significant increase in IOP after DEX implants compared to anti-VEGF treatment, which agreed with another meta-analysis that compared DEX implants and anti-VEGF treatment related to diabetic retinopathy [36]. But the current results found little difference between these two treatments. As CRVO can be divided into ischaemic and non-ischaemic types, this minor difference in IOP may be caused by the different natural course and varying extent of primary diseases. Moreover, our meta-analysis showed a higher risk of cataracts after DEX implants, suggesting that ophthalmologists should pay attention when using DEX implants in patients with a clear lens. Since cataracts can influence VA, studies of RVO patients with pseudophakic eyes should be conducted.

Due to the limited number of reports included in this study, several quantitative results presenting high heterogeneity and other heterogeneity test methods including funnel plots were not conducted. Feltgen et al.’s research was actually the extension of previous BRVO and CRVO studies. In addition to the different observation time intervals and varying treatment approaches within these included studies, the current quantitative synthesis neglecting origin could lead to measurement bias.

Recently few studies have concentrated on the PRN treatment of DEX implants, and different anti-VEGF treatments could lead to varying results. In the future, comparisons related to functional improvements, adverse effects, and economic costs between DEX implants and anti-VEGF treatment under both PRN models should be conducted to draw more precise conclusions. In the meantime, studies comparing different anti-VEGF agents to DEX implants for longer observation time intervals should be conducted. Moreover, as CRVO can be divided into ischaemic and non-ischaemic types and BRVO can present varied clinical manifestations when different branch retinal veins are involved, more multi-centre RCTs with accurate subdivisions should be implemented in the future.

## Conclusions

In summary, this meta-analysis of four RCTs relating to RVO treatments revealed that anti-VEGF treatment presented better functional and anatomical improvements than DEX implants, which was achieved by the 3 + PRN model in anti-VEGF treatment. In addition, DEX implants presented significant higher risk of IOP elevation and cataract forming compared to anti-VEGF treatment. In the future, studies assessing DEX implants under the PRN model as well as the subdivision of RVO should be conducted. In addition, new treatment methods such as combined therapy should be developed and investigated to optimise clinical efficacy, economic cost, and side effects.

## Abbreviations

BCVA: Best-corrected visual acuity
BRVO: Branch retinal vein occlusion
CI: Confidence intervals
CRVO: Central retinal vein occlusion
CST: Central subfield thickness
DEX: Dexamethasone
ETDRS: Early Treatment Diabetic Retinopathy Study
IOP: Intraocular pressure
MD: Mean differences
OR: Odds ratios
PRN: Pro re nata
RCT: Randomised controlled trial
RVO: Retinal vein occlusion
SD: Standard deviation
SE: Standard error
VA: Visual acuity
VEGF: Vascular endothelial growth factor
